# Invasive clear-cell variant of squamous cell carcinoma mimicking sebaceous carcinoma^⋆^

**DOI:** 10.1016/j.abd.2022.05.007

**Published:** 2023-04-20

**Authors:** Emilly Neves Souza, Lucia Martins Diniz, Luana Amaral de Moura, Alexandre Calegari Oliosi, Marcela Scárdua Sabbagh de Azevedo, Márgya Neves Souza

**Affiliations:** a Department of Dermatology, Hospital Universitário Cassiano Antônio Moraes, Universidade Federal do Espírito Santo, Vitória, ES, Brazil; b Faculdade Multivix, Vitória, ES, Brazil

Dear Editor,

A Caucasian woman, 54 years old, Fitzpatrick phototype II, complained of a painful lesion on the right malar region, with rapid growth and aggressive behavior, which had appeared three months before. The patient lived in a rural area and worked in agriculture, referring chronic sun exposure due to her working conditions. When questioned, she denied history of trauma at the site of the lesion, previous skin cancer, immunosuppression, and exposure to artificial radiation. Dermatological examination showed an infiltrated and painful erythematous plaque with exudation, crusts, ulceration, and telangiectasias in the malar region extending to the right nasal ala (Fig. 1). Skin damage due to chronic sun exposure was also identified, manifesting as xerosis, melanoses, solar elastosis, actinic keratoses, and loss of skin elasticity on the face, anterosuperior thoracic region, and upper limbs. The initial diagnostic hypotheses were terebrant basal cell carcinoma and invasive squamous cell carcinoma. An incisional biopsy of the lesion was performed and histopathology showed a malignant epithelial proliferation with islands of clear cells and areas of necrosis; in addition to some cells with vacuolated cytoplasm, suggestive of a sebaceous neoplasia (Fig. 2). Rare keratinization foci were identified, suggesting squamous cell carcinoma (Fig. 3). Periodic acid Schiff (PAS) staining with and without diastase revealed the presence of cytoplasmic glycogen, excluding the possibility of sebaceous differentiation.

**Figure 1:**
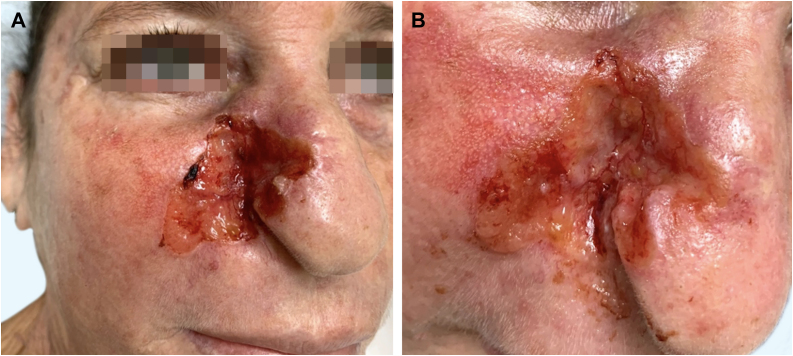
(A) Infiltrated erythematous plaque with exudation, crusts and ulceration in the right malar region, on photodamaged skin. (B) Exuberant vascular proliferation extending to the right nasal ala

**Figure 2:**
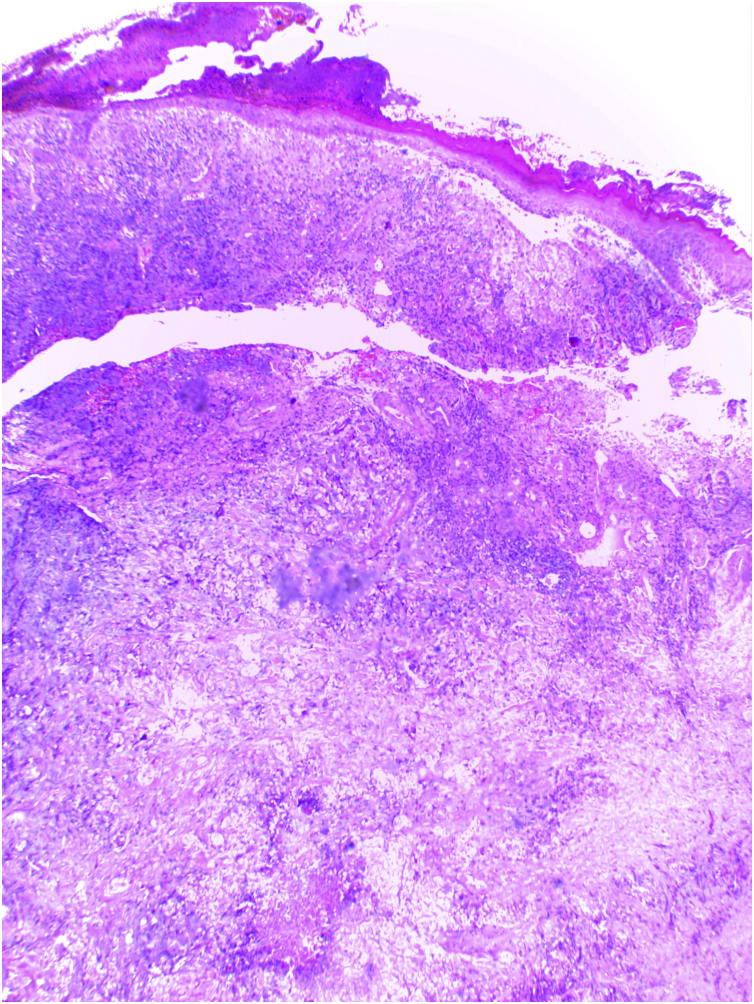
Malignant epithelial proliferation, with islands of clear cells and areas of necrosis. Some cells have vacuolated cytoplasm (Hematoxylin & eosin, ×100)

**Figure 3:**
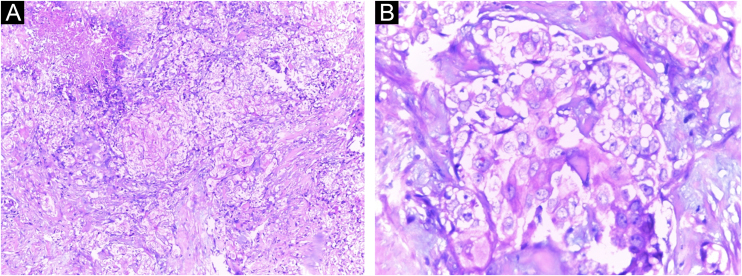
(A) Squamous cell carcinoma with clear cell differentiation, showing cells with clear to vacuolated cytoplasm (Hematoxylin & eosin, ×200). (B) Clear cells in the middle of the squamous differentiation area, with rare keratinization foci (Hematoxylin & eosin, ×400)

The immunohistochemical analysis showed positivity for cytokeratin 5/6 and p63; and was focally positive for BerEp4, EMA, and factor XIIIa; with negativity for adipophilin, androgen receptor and CEA (Fig. 4), confirming the squamous origin of the neoplasm. Face and neck computed tomography showed a solid expansive lesion with extensive superficial ulceration and intense heterogeneous contrast enhancement, extending to the right nasal ala and maxillary region. Nasal and right maxillary sinus bone destruction was observed, close to the right orbit and the dental root of the upper right canine (Fig. 5). No metastases were found in the imaging studies. Considering the extent of the lesion, the oncology and head and neck surgery team opted for neoadjuvant chemotherapy and subsequent evaluation of a surgical approach.

**Figure 4:**
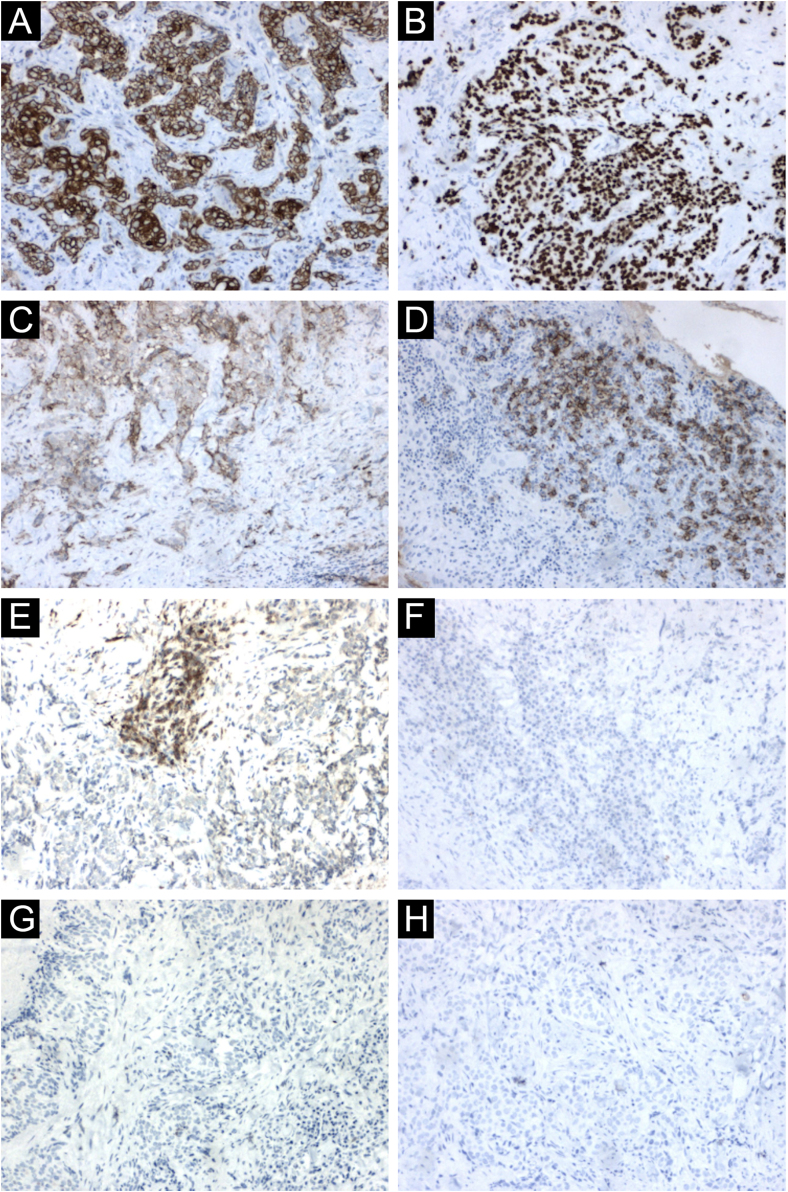
Immunohistochemical findings. (A) Neoplastic cells immunoreactive with cytokeratin 5/6. (B) Neoplastic cells immunoreactive with p63. (C) Focal positivity for BerEp4. (D) Focal positivity for EMA. (E) Focal positivity for factor XIIIa. (F) Neoplastic cells non reactive with adipophilin, (G) with androgen receptor (H) and with CEA

**Figure 5:**
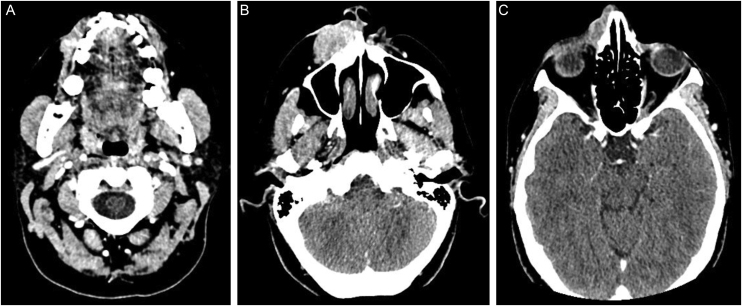
Computed tomography of the face and neck: (A) proximity of the neoplasm to the dental root of the upper right canine. (B) solid expansive lesion with wide superficial ulceration, extending to the nasal ala and right maxillary region, measuring 3.6 × 1.9 × 4.5 cm. (C) nasal bone destruction and upper extension, close to the right orbit. The tumor shows marked and heterogeneous contrast enhancement

## Discussion

Cutaneous squamous cell carcinoma (SCC) is classified into many subtypes, with a wide range of clinical manifestations ranging from indolent growth to aggressive tumors with significant metastatic potential.^1^ The undetermined category includes signet-ring SCC, follicular SCC, papillary SCC, SCC arising in adnexal cysts, squamoid eccrine ductal carcinoma, and SCC with clear-cell differentiation.^1^

Cutaneous clear-cell SCC is a rare neoplasia the etiology of which is still incompletely understood. This variant was described in 1980 by Kuo.^2^ Since then, around ten articles have been published about this neoplasm,^2^, ^3^, ^4^, ^5^, ^6^, ^7^, ^8^, ^9^ none of them in Brazil (Table 1).

**Table 1:** Summary of published cases of invasive cutaneous squamous cell carcinoma with clear cell differentiation

Author/Year	Age (years)/Sex	Profession	Lesion location	Time of evolution	Clinical description	Treatment	Follow-up
Kuo (1980)^2^	75, M	Mechanic	Right malar region	Several months	1 × 1.4 cm, ulcerated nodule	Excision	No recurrence after 22 months
	65, F	Operator	Left jaw	2 months	2 × 3 cm, painful mass	Hemimandibulectomy	Death after one year due to extensive local recurrence
						Radical neck dissection	
						Radiotherapy	
	70, M	Agricultural worker	left jaw angle	2 months	2 cm, raised painless mass	Excision	Recurrence and re-excision after three months. No further recurrence after 21 months.
	80, M	Agricultural worker	Right jaw angle	4 months	1.5 × 2 cm raised mass	Steroid injection	No recurrence after 22 months
						Excision	
						Radiotherapy	
	52, M	Soccer coach	Left jaw angle	4 months	4 × 5 mm, painless mass	Local excision	No recurrence after 33 months
						Parotidectomy	
						Radical neck excision	
	72, M	Farm inspector	Left cervical region	10 months	Ulcerated, extensive, friable mass in the left cervical region	Incomplete excision	Postoperative death due to pneumonia
Requena et al. (1991)^3^	62, M	‒	Right malar region	6 months	2 cm, nodule	Excision	‒
Corbalán-Vélez et al. (2009)^4^	7 patients (M and F)	No specific individual information					
Lawal et al. (2013)^6^	62, M	Bricklayer	Left facial region	6 months	16 cm, large exophytic mass	Patient chose not to undergo treatment	‒

F, Female; M, Male.

SCC usually manifests itself on photo exposed areas of elderly Caucasian men with a history of chronic sun exposure and multiple skin neoplasms.^2^, ^6^, ^8^ Most articles in the literature describe head and neck lesions, some of which with rapid growth.^8^ In the present case, a rapid-growing lesion with aggressive behavior was identified on the face of a 54-year-old woman, with a history of chronic sun exposure and no previous skin neoplasia.

Clear-cell SCC can be classified into three variants: keratinizing (type I), non-keratinizing with no connection to the epidermis (type II), and pleomorphic (type III).^2^ The histopathological findings of the present report are consistent with the keratinizing variant (type I) according to Kuo.^2^ In 2007, a new classification was proposed according to the number of clear cells in the anatomopathological evaluation: cases with ≥80% of clear cells represent clear-cell SCC; cases with <80% and ≥50% are defined as SCC with marked clear cell change; and cases with <50% and >10% are described as SCC with moderate clear cell change.^5^ According to this classification, the neoplasm described in the present case can be identified as SCC with marked clear cell change.

On histopathology, the atypical clear cells showed evident nuclear pleomorphism, squamous differentiation foci, and areas of acantholysis, with some dyskeratotic cells amidst pseudo glandular spaces.^1^ The main differential diagnosis is sebaceous carcinoma, which is characterized by vacuolated, lipid-containing cytoplasmic cells positive for factor XIIIa, EMA, adipophilin, androgen receptor, AE1/AE3 cytokeratins, and perilipin on immunohistochemical analysis.^1^, ^10^ In the present case report, the immunohistochemical analysis disclosed marked positivity for cytokeratin 5/6 and p63, with focal positivity for BerEp4, EMA, and factor XIIIa. Adipophilin and androgen receptors were non reactive (Fig. 4). Other differential diagnoses include trichilemmoma, clear cell acanthoma, pilar tumor, balloon cell nevus, balloon cell melanoma, and renal metastatic carcinoma.^6^ Mohs micrographic surgery is the treatment of choice for head and neck cutaneous SCC.

It is difficult to define the prognosis of this rare variant, as few cases were published in the literature up to 2022. Cohen et al. (2008) suggested that human papillomavirus (HPV) infection may be associated with tumor oncogenesis, but only two reported cases were associated with this virus.^9^ New studies are required to elucidate the behavior of clear cell SCC.

In conclusion, the present study describes the atypical case of a 54-year-old woman with a facial neoplasm compatible with clear cell SCC, histopathologically mimicking sebaceous carcinoma. Both dermatologists and dermatopathologists must be aware of the peculiarities of this SCC variant, which grew fast and was quite aggressive in the present report.

## Financial support

None declared.

## Authors' contributions

Emily Neves Souza: Design and planning of the study; drafting and editing of the manuscript; collection, analysis, and interpretation of data; critical review of the literature; critical review of the manuscript.

Lucia Martins Diniz: Design and planning of the study; effective participation in research orientation; intellectual participation in the propaedeutic and/or therapeutic conduct of the studied case; critical review of the literature; critical review of the manuscript; approval of the final version of the manuscript.

Luana Amaral de Moura: Design and planning of the study; drafting and editing of the manuscript; collection, analysis, and interpretation of data; critical review of the literature.

Alexandre Calegari Oliosi: Design and planning of the study; drafting and editing of the manuscript; collection, analysis, and interpretation of data; critical review of the literature.

Marcela Scárdua Sabbagh de Azevedo: Design and planning of the study; drafting and editing of the manuscript; collection, analysis, and interpretation of data; critical review of the literature.

Márgya Neves Souza: Design and planning of the study; drafting and editing of the manuscript; collection, analysis, and interpretation of data; critical review of the literature.

## Conflicts of interest

None declared.
